# Supplementary material for: Non-stationary coherent quantum many-body dynamics through dissipation
Source: Nat Commun. 2019 Apr 15;10:1730. doi: 10.1038/s41467-019-09757-y (PMC6465298; doi:10.1038/s41467-019-09757-y)
Supplement: Supplementary file 1 — Supplementary Information [file 41467_2019_9757_MOESM1_ESM.pdf]

Supplementary Information: Non-stationary coherent quantum many-body dynamics through dissipation

Buča, et al.

Supplementary Methods

Introduction

Let us recall the Lindblad equation,

$$\frac{\mathrm{d}}{\mathrm{d}t}\rho(t) = \mathscr{L}\rho(t) := \\ -\mathrm{i}[H,\rho(t)] + \sum_{\mu}\left(2L_{\mu}\rho(t)L_{\mu}^{\dagger} - \{L_{\mu}^{\dagger}L_{\mu},\rho(t)\}\right), \tag{1}$$

and the eigenvalue equation for the Liouvillian,

$$\mathscr{L}\rho = \lambda\rho \iff \mathscr{L}\rho^{\dagger} = \lambda^{*}\rho^{\dagger}. \tag{2}$$

We set $\hbar = 1$ for simplicity of notation. We are concerned with cases where λ is purely imaginary. The corresponding eigenmodes are called oscillating coherences,[1] or limit cycles.[2] Here we emphasize that our results may be also understood as a framework for constructing genuine *many-body* quantum synchronization.[2–4]

Formal classification of the asymptotic subspaces and relation to decoherence-free subpaces

To make connection with the existing literature we will discuss the formal classification of the asymptotic subspace of the Liouvillian that we constructed here.

We will follow the terminology of Supplementary Reference 5 (see also Supplementary Referece 6). The asymptotic subspaces of the quantum Liouvillian from the main text, that the dynamics is leads to in the long-time limit, is a *multi-block* structure, which is the most general possible form that it can have.[5]

More specifically, for the Hubbard model example in the main text, by indexing the subspaces of $S^{+}S^{-}$ (N) as μ (ν) (where S^{α} are the total spin operators and N is the total particle number) the basis of the asymptotic subspace can be written in the form,

$$|z_1^{\mu,\nu}\rangle\langle z_2^{\mu,\nu}| \otimes P_{\mu}^{S^{+}S^{-}} P_{\nu}^{N}, \tag{3}$$

where $P_{\mu}^{S^{+}S^{-}}$ and P_{ν}^{N} are projectors to the corresponding subspaces of $S^{+}S^{-}$ and N, respectively, and $|z_{1,2}^{\mu,\nu}\rangle$ are the corresponding eigenstates of S^{z}.

The form of the multi-block of the other examples featuring the Hubbard model, discussed later in in this supplementary, are more difficult to construct, due to the fact that the corresponding quantum Liouvillian will not be unital (i.e. $\mathscr{L}\mathbb{1} \neq 0$).

We now further remark on the differences between this type of structure and a dynamical decoherence-free subspace widely studied in literature (for references beyond the ones cited in the main text see e.g. Supplementary References 7–9). A decoherence-free subspace is a subspace of the Hilbert space invisible to dissipation, i.e. $\mathscr{H}_{DFS} \subseteq \mathscr{H}$ and for any pure state $|\psi(t)\rangle \in \mathscr{H}_{DFS}$ (with $\rho(t) = |\psi(t)\rangle\langle\psi(t)|$) we have $\partial_t \mathrm{tr}\rho(t)^2 = 0$ (e.g. Def. 2 of Supplementary Reference 7). In other words, all pure states in the subspace undergo coherent time evolution given by the system's Hamiltonian and remain pure.[5] Therefore, they may be understood as restriction of the closed system's Hamiltonian to a subspace to which the dissipative time evolution guides the system in the long time limit. For instance, the open XXZ spin ring later in this supplementary material is a decoherence-free subspace. This open XXZ spin ring is, to our knowledge, the first example of a decoherence-free subspace in an open quantum *many-body* system.

In contrast to a decoherence-free subspace the multi-block structure, for which we provide sufficient criteria, is affected by the dissipation. The asymptotic dynamics is coherent, but it consists of generally mixed states. The physical properties of these mixed states are affected by the dissipation. For instance, the dissipation may induce off-diagonal long-range order (like in the example in the main text), or currents of some quantity (like in the examples in the later sections). Furthermore, when we study the quantum stochastic process[10] corresponding to the master equation given in Supplementary Equation (1), the dynamics in a decoherence-free subspace is purely deterministic (every quantum trajectory is the same, like in the closed system), whereas in the multi-block structure the dynamics is stochastic (the ensemble of trajectories is non-trivial).

Generalizing beyond the Markovian framework

It is fairly straightforward to generalize the discussion in the main text beyond the Markovian framework. Let the full Hamiltonian of the system (with Hilbert space $\mathscr{H}_S$) and bath (with Hilbert space $\mathscr{H}_B$) be given as

$$H = H_S + H_{SB} + H_B, \tag{4}$$

where $H_S \in \mathscr{H}_S$ is the system's Hamiltonian, $H_B \in \mathscr{H}_B$ is the Hamiltonian of the bath and $H_{SB} \in \mathscr{H}_S \otimes \mathscr{H}_B$ is the system-bath interaction. In general, we may write $H_{SB} = \sum_j S_j \otimes B_j$, where $S \in \mathscr{H}_S$ and $B \in \mathscr{H}_B$.

Let A be an eigenoperator of the full Hamiltonian, $[A,H] = \lambda A$. As H is Hermitian $\lambda \in \mathbb{R}$. The case studied in the main text would correspond to $A = A_S \otimes \mathbb{1}_B$, with $A_S \in \mathscr{H}_S$ and,

$$[A_S, H_S] = \lambda A_S, \qquad [A_S, H_{SB}] = 0. \tag{5}$$

The full time evolution of the open system is given as,

$$\rho_S(t) := \hat{\mathscr{T}}_t \rho(0) = \mathrm{tr}_B \left[e^{-\mathrm{i}Ht} \rho(0) e^{\mathrm{i}Ht} \right], \tag{6}$$

where tr_B represents tracing over the bath degrees of freedom.

Let $\rho_\infty \in \mathscr{H}_S$ be a stationary state of the dynamical map in the sense that for some $\rho'(0)$ we have,

$$\rho_\infty = \lim_{t\to\infty} \hat{\mathscr{T}}_t \rho'(0). \tag{7}$$

Such as state always exists (e.g. any incoherent mixture of the eigenstate of H will produce such a state trivially). We look at the asymptotic time evolution of $\rho_{nm} = A^n \rho'(0)(A^\dagger)^m$. It follows from $[H,A] = \lambda A$ that,

$$e^{-\mathrm{i}Ht} A e^{\mathrm{i}Ht} = e^{\mathrm{i}\lambda t} A. \tag{8}$$

We decompose the density matrix of the full system and bath as $\rho(t) = \sum_{i,j} s_i(t) \otimes b_j(t)$ and write,

$$\rho_S(t) = \mathrm{tr}_B \sum_{i,j} s_i(t) \otimes b_j(t) = \sum_{i,j} c_j(t) s_i(t), \tag{9}$$

where $s_j(t) \in \mathscr{H}_S$ and $b_j(t) \in \mathscr{H}_b$ and $c_j(t) = \mathrm{tr}_B b_j(t)$.

Then,

$$\begin{aligned}
\lim_{t\to\infty} \hat{\mathscr{T}}_t \rho_{nm} &= \lim_{t\to\infty} \mathrm{tr}_B \left[e^{-\mathrm{i}Ht} A^n \rho'(0)(A^\dagger)^m e^{\mathrm{i}Ht} \right] \\
&= \lim_{t\to\infty} \mathrm{tr}_B \left[e^{\mathrm{i}n\lambda t} A^n e^{-\mathrm{i}Ht} \rho'(0) e^{\mathrm{i}Ht} (A^\dagger)^m e^{-\mathrm{i}m\lambda t} \right] \\
&= \lim_{t\to\infty} \mathrm{tr}_B \left[e^{\mathrm{i}n\lambda t} A^n \left(\sum_{i,j} s_i(t) \otimes b_j(t) \right) (A^\dagger)^m e^{-\mathrm{i}m\lambda t} \right] \\
&= \lim_{t\to\infty} e^{\mathrm{i}n\lambda t} A^n \left(\sum_{i,j} c_j(t) s_i(t) \right) (A^\dagger)^m e^{-\mathrm{i}m\lambda t} \\
&= \lim_{t\to\infty} e^{\mathrm{i}n\lambda t} A^n \rho_\infty (A^\dagger)^m e^{-\mathrm{i}m\lambda t},
\end{aligned} \tag{10}$$

where to obtain the second equality we repeatedly inserted $\mathbb{1} = e^{-\mathrm{i}Ht} e^{\mathrm{i}Ht}$ between the products of A's and used Supplementary Equation (8). To obtain the third and fourth equality we used Supplementary Equation (9) and the fact that $A \in \mathscr{H}_S$. The last equality was obtained by recognizing that $\rho_\infty = \lim_{t\to\infty} \rho_S(t)$ is a stationary state.

We emphasize that ρ_{nm} are *not* density matrices, but their linear combinations can be chosen to be.

Dynamical decoherence-free many-body subspaces

We will define what we mean by a *standard* dark Hamiltonian $\mathfrak{H} := [\tilde{H}, \bullet]$ (or dynamical many-body decoherence-free subspace) with $\tilde{H} = \tilde{H}^\dagger$. We want the spectrum of $\mathfrak{H}$ to be made up of *pure* mutually orthogonal eigenstates and to have the same left and right eigenvectors. Namely:

1. We first wish that,

$$\mathfrak{H} |\phi_n\rangle\langle\phi_m| = (\omega_n - \omega_m) |\phi_n\rangle\langle\phi_m|, \quad \omega_k \in \mathbb{R}, \forall k. \tag{11}$$

2. Where we also desire that eigenmodes $\rho_{nm} := |\phi_n\rangle\langle\phi_m|$ are mutually orthogonal in the Hilbert-Schmidt sense, $\mathrm{tr}\rho_{nm}^{\dagger}\rho_{n'm'} = \delta_{n,n'}\delta_{m,m'}$.

3. Finally we require that the left and right eigenvectors match ensuring that $\mathfrak{H} = \mathfrak{H}^{\dagger}$.

We state the details of the conditions under which properties 1-3 will be fulfilled in Supplementary Theorem 1.

Supplementary Theorem 1. *A set of mutually orthogonal vectors* $\{|\phi_1\rangle, |\phi_2\rangle, \ldots\}$ *forms a set of eigenvectors of a standard dark Hamiltonian (or a decoherence-free subspace) in the sense of properties 1-3 iff the following conditions are fulfilled,*

(a) $\left(\mathrm{i}H + \sum_k \gamma_k L_k^{\dagger} L_k\right)|\phi_n\rangle = \lambda_n |\phi_n\rangle, \quad \forall n,$

(b) $L_k |\phi_n\rangle = \lambda_{k,n} |\phi_n\rangle$, *and* $\sum_k \gamma_k |\lambda_{k,n}|^2 = \mathrm{Re}\,\lambda_n, \quad \forall n,$

(c) $\mathrm{Re}\left[\sum_k \gamma_k \left(2\lambda_{k,n}\lambda_{k,m}^{*} - |\lambda_{k,n}|^2 - |\lambda_{k,m}|^2\right)\right] = 0, \forall n, m.$

Then the eigenvalues from Supplementary Equation (11) *are given as*

$$\omega_n - \omega_m = \mathrm{Im}\left(\sum_k 2\gamma_k \lambda_{k,n}\lambda_{k,m}^{*} - \langle\phi_m|H|\phi_n\rangle\right). \tag{12}$$

Proof. Conditions (a) and (b) are just the well-known conditions of Theorem 1 of Supplementary Reference 11 for pure stationary states $\mathscr{L}(|\phi_n\rangle\langle\phi_n|) = 0$. Condition (c) can be shown as follows: We begin by writing out Supplementary Equation (2) for $\rho = |\phi_m\rangle\langle\phi_n|$, and taking the product with $\langle\phi_m|$ from the left and $|\phi_n\rangle$ from the right. Then we demand that the corresponding eigenvalue λ is purely imaginary and use conditions (a) and (b) of the theorem. Recalling the definitions in Supplementary Equation (11), this also leads to Supplementary Equation (12). □

Supplementary Theorem 2. *If there is no subspace* $\mathscr{S} \subset \mathscr{H}$ *that is orthogonal to the space of dark states*[1] $\mathscr{D}$ *(i.e.,* $\mathscr{S} \perp \mathscr{D}$*) such that* $L_k\mathscr{S} \subset \mathscr{S}$*, then the only oscillating coherences are vectors in a decoherence free subspace in the sense that they have the form* $|\phi_n\rangle\langle\phi_m|$ *with* $|\phi_i\rangle \in \mathscr{D}$ *being dark states.*

Proof. Theorem 2 of Supplementary Reference 11 guarantees that if there is no such subspace $\mathscr{S}$ then the only stationary states are dark states $\mathscr{L}|\phi_n\rangle\langle\phi_n| = 0$. According to Theorem 18 of Supplementary Reference 6 all oscillating coherences must be of the form $A\rho_\infty$ where $\mathscr{L}\rho_\infty = 0$ and A is an operator. We know that the only stationary states are of the form $|\phi_n\rangle\langle\phi_n|$. Writing $|\Psi\rangle = A|\phi_n\rangle$, we have that any

[1] Dark states $|\phi_n\rangle$ are defined as $L_k|\phi_n\rangle = 0$ and $H|\phi_n\rangle = \omega_n|\phi_n\rangle$, $\forall k$.

oscillating coherence must satisfy
$-\mathrm{i}[H,|\Psi\rangle\langle\phi_n|]+\sum_\mu \gamma_\mu \left(2L_\mu|\Psi\rangle\langle\phi_n|L_\mu^\dagger-\{L_\mu^\dagger L_\mu,|\Psi\rangle\langle\phi_n|\}\right)=\mathrm{i}\Lambda|\Psi\rangle\langle\phi_n|$, with $\Lambda\in\mathbb{R}$. Using $L_k|\phi_n\rangle=0$ and $H|\phi_n\rangle=\omega_n|\phi_n\rangle$ we are left with $-\mathrm{i}H|\Psi\rangle\langle\phi_n|-L_\mu^\dagger L_\mu|\Psi\rangle\langle\phi_n|=\mathrm{i}\Lambda|\Psi\rangle\langle\phi_n|$. We take the product of this with $\langle\Psi|$ from the left and $|\phi_n\rangle$ from the right, thus we have $\langle\Psi|L_k^\dagger L_k|\Psi\rangle=||L_k|\Psi\rangle||^2\geq 0$. We then use the fact that H is Hermitian and so $\langle\Psi|H|\Psi\rangle\in\mathbb{R}$. Then the only way to satisfy the eigenequation is iff $L_k|\Psi\rangle=0$ and $H|\Psi\rangle=\Lambda|\Psi\rangle$, therefore $|\Psi\rangle\in\mathscr{D}$ is also a dark state. □

Theorems on general complex coherent dynamics under dissipation

In this section we go beyond $\mathfrak{H}$ being a standard Hamiltonian, i.e. $\mathfrak{H}\neq[\tilde{H},\bullet]$, and move to cases when the eigenmodes of $\mathfrak{H}$ are not pure states and when $\mathfrak{H}$ is not Hermitian.

Supplementary Theorem 3. *Let $\mathscr{L}\rho_\infty=0$. If there exists a non-trivial operator A with the property*

$$[H,A]\rho_\infty=\lambda A\rho_\infty,\quad [L_k,A]\rho_\infty=[L_k^\dagger,A]L_k\rho_\infty=0,\quad \forall k, \tag{13}$$

then the state $\rho=A\rho_\infty$ is an eigenstate of the Liouvillian with purely imaginary eigenvalue, $\mathscr{L}\rho=\mathrm{i}\lambda\rho,\lambda\in\mathbb{R}$.

Proof. Take the conjugate transpose of Supplementary Equation (13). We get $\rho_\infty[H,A^\dagger]=-\lambda^*\rho_\infty A^\dagger$ and $\rho_\infty[L_k^\dagger,A^\dagger]=\rho_\infty L_k^\dagger[L_k,A^\dagger]=0,\forall k$, as ρ_∞ is Hermitian. Now define a superoperator $\hat{S}\rho:=[H,A^\dagger A]\rho,\forall\rho\in\mathrm{End}(\mathscr{H})$. The superoperator is a commutator of two Hermitian operators and is therefore clearly skew-Hermitian (with purely imaginary eigenvalues). We take the Hilbert-Schmidt inner product of this superoperator with ρ_∞. The result is purely imaginary, $\mathrm{tr}\left(\rho_\infty[H,A^\dagger A]\rho_\infty\right)=\mathrm{i}\theta,\theta\in\mathbb{R}$, as $\hat{S}$ is skew-Hermitian. It is straightforward to calculate, using Supplementary Equation (13) and the conjugate transpose of Supplementary Equation (13), that $\mathrm{tr}\left(\rho_\infty\hat{S}\rho_\infty\right)=(\lambda-\lambda^*)\mathrm{tr}\left(\rho_\infty A^\dagger A\rho_\infty\right)$. However, $\mathrm{tr}\left(\rho_\infty A^\dagger A\rho_\infty\right)=||A\rho_\infty||>0$. Thus the rhs is purely real, whereas the lhs is purely imaginary. Therefore, $\lambda=\lambda^*$ (i.e, $\lambda\in\mathbb{R}$).

We define a new superoperator $\hat{A}\rho:=A\rho,\forall\rho\in\mathrm{End}(\mathscr{H})$. Using the definition of the Liouvillain $\mathscr{L}$ from Supplementary Equation (1) and using Supplementary Equation (13) (and its conjugate transpose), it is straightforward to show that $[\mathscr{L},\hat{A}]\rho_\infty=\mathrm{i}\lambda\hat{A}\rho_\infty$. Then, using $\mathscr{L}\rho_\infty=0$, the statement of the theorem directly follows. □

Supplementary Corollary 1. *In particular, if*

$$[H,A]=\lambda A,\quad [L_k,A]=[L_k^\dagger,A]=0,\quad \forall k, \tag{14}$$

then $\mathscr{L}\rho_{nm}=\mathrm{i}\lambda(n-m)\rho_{nm},\lambda\in\mathbb{R}$, with $\rho_{nm}=A^n\rho_\infty(A^\dagger)^m$.

Proof. From Supplementary Equation (14) it also follows that $[H,A^n]=n\lambda A^n,[L_k,A^n]=[L_k^\dagger,A^n]=0,\forall k,n$. Taking the conjugate transpose of that we have $[H,(A^\dagger)^n]=-n\lambda(A^\dagger)^n$, $[L_k,(A^\dagger)^n]=[L_k^\dagger,(A^\dagger)^n]=0,\forall k,n$.

Assume without loss of generality that $n > m$: then it follows from Supplementary Equation (14), its conjugate transpose and Supplementary Equation (1) that $\mathscr{L}A^m\rho_\infty(A^\dagger)^m = 0$, i.e. $\rho'_\infty = A^m\rho_\infty(A^\dagger)^m$ is also a stationary state. Define $A' = A^{n-m}$, we then have $\rho_{nm} = A'\rho'_\infty$ and we can now simply invoke Supplementary Theorem 3 and arrive at the main statement of the corollary. Recalling that it also directly follows that the state $\rho^\dagger_{nm}$ is an eigenstate with an eigenvalue of opposite sign $\mathscr{L}\rho^\dagger_{nm} = -\mathrm{i}(n-m)\lambda\rho^\dagger_{nm}, \lambda \in \mathbb{R}$, we can repeat the same procedure assuming that $m > n$. This proves the corollary. □

Supplementary Discussions

We now proceed to give examples and discuss the proposed experimental implementations.

Dynamical decoherence-free subspace: The XXZ spin ring

We study the n-site Heisenberg XXZ spin chain with periodic boundary conditions (spin ring),

$$H_{\mathrm{XXZ}} = \sum_{j=1}^{n} \sigma^+_j\sigma^-_{j+1} + \sigma^-_j\sigma^+_{j+1} + \Delta\sigma^{\mathrm{z}}_j\sigma^{\mathrm{z}}_{j+1}, \tag{15}$$

where Δ is an anisotropy parameter and the spin-$1/2$ operators on site j are $\sigma^\alpha_j = \mathbb{1}_2^{\otimes(j-1)} \otimes \sigma^\alpha \otimes \mathbb{1}_2^{\otimes(n-j)}$ (with σ^α being the standard Pauli matrices and $\mathbb{1}_2$ is the identity matrix of size 2x2). For the sake of simplicity, we study the dimensionless version of the model. We also introduce a single ultra-local loss term $L = \gamma\sigma^-_1$ with loss rate $\gamma \geq 0$. In the long-time limit this setup induces a dynamical decoherence-free many-body subspace (see Supplementary Theorems 1 and 2) that is formed from pure states that are eigenstates of the Hamiltonian $H_{\mathrm{XXZ}}|\phi_n\rangle = \omega_n|\phi_n\rangle$ and that are annihilated by $L|\phi_n\rangle = 0$. Thus, $\mathscr{L}|\phi_n\rangle\langle\phi_m| = \mathrm{i}(\omega_n - \omega_m)|\phi_n\rangle\langle\phi_m|$, and any state of the form $|\Psi\rangle = |\phi_n\rangle + e^{\mathrm{i}\alpha}|\phi_m\rangle$ will undergo oscillations when $\omega_n \neq \omega_m$, $\forall\alpha$.

The Liouvillian of the model was studied through exact diagonalization, and by requiring that the Bethe ansatz solutions for the eigenvectors of H_{XXZ} are dark states of L. The spectrum of the corresponding Liouvillian is shown in Supplementary Figure 1 for different system sizes n. We find that the number of distinct purely imaginary eigenvalues scales sub-quadratically with n. The spectrum in Supplementary Figure 1 also shows the formation of oscillatory patterns in the spectral densities that hint at the formation of Bethe strings in the values of the complex quasi-rapidities of the related integrable model. The eigenstates with purely imaginary eigenvalues are states with nodes on the loss site (and may also be understood as lattice scarring,[12] but a 1D quantum many-body version[13]). The purely imaginary eigenvalues are not present at the non-interacting point of the model ($\Delta = 0$).

In the thermodynamic limit the spectrum of the lossy XXZ spin ring Liouvillian seems to be incommensurate. This may again lead to relaxation, though it would likely require much longer time than

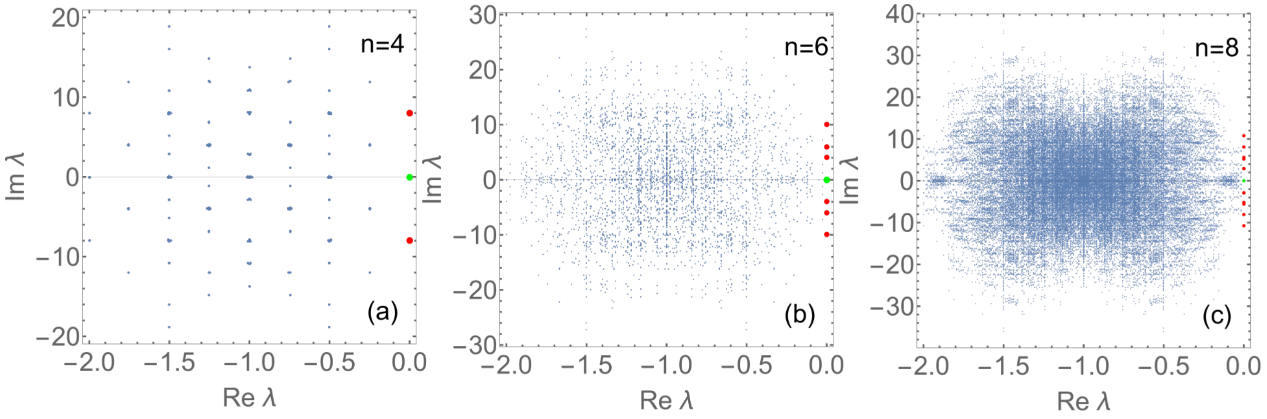

Supplementary Figure 1: The spectrum of the XXZ Liouvillian with a single loss term. We study three system sizes n=4 (a), n=6 (b) and n=8 (c) in the gapped regime with $\Delta = 2$ and $\gamma = 1$ and plot the real and imaginary part of the eigenvalues of $\mathscr{L}$. The larger red points indicate the purely imaginary eigenvalues and the green points the stationary states (with eigenvalue 0).

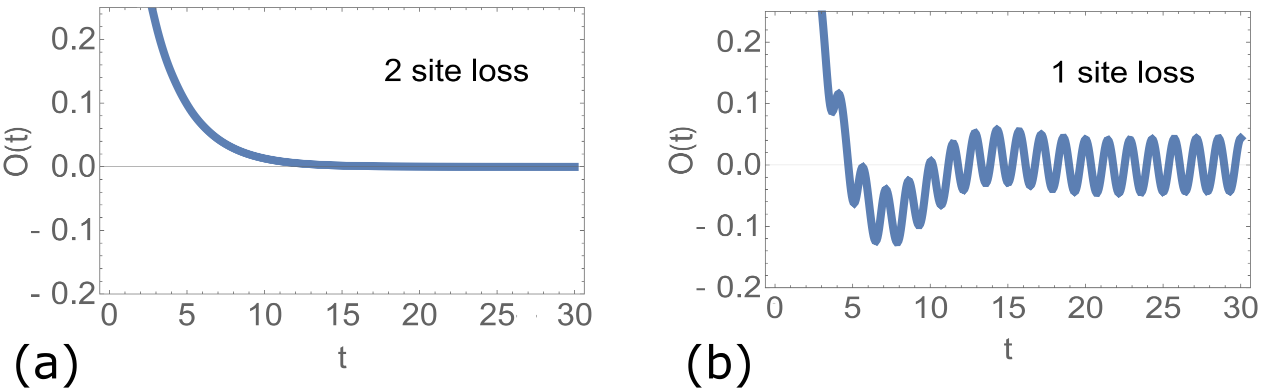

Supplementary Figure 2: Comparison of the dynamics of the XXZ spin ring for $\Delta = 1.1$, $n = 4$, $\gamma = 1$ with two (a) and one (b) loss terms. Introducing two loss terms destroys the dynamical decoherence-free subspace and leads to relaxation to stationarity (a). One loss term, instead, preserves this dynamical subspace and leads to persistent oscillations. Results shown for $O(t) = \langle \sigma_2^x(t) \rangle$.

in the corresponding closed XXZ ring

In the long-time limit this setup restricts the dynamics to the dynamical decoherence-free many-body subspace that undergoes continued oscillations. We take $\Delta = 1.1$, $n = 4$, $\gamma = 1$ and show the dynamics in Supplementary Figure 2 for $O(t) = \langle \sigma_2^x(t) \rangle$. The time evolution is initialized in a random initial state. Numerical investigation indicates that the dynamical decoherence-free subspace is robust to terms in the Hamiltonian that break integrability, but it is not robust to the presence of additional Lindblad operators. In particular, adding a second loss term destroys the dynamical decoherence-free subspace. We thus compare the time evolution of the same observable starting in the same initial state and with the same parameters, but with two loss terms $L_1 = \gamma_1 \sigma_1^-$, $L_2 = \gamma_2 \sigma_2^-$ ($\gamma_1 = \gamma_2 = \gamma$) in Supplementary Figure 2. This case clearly shows relaxation to stationarity. Furthermore, the stationary state is unique.

Multi-block structures: Additional examples with the Hubbard model

Spin dephasing

Due to the spin and η-pairing symmetries of the D-dimensional Hubbard model the conditions of Supplementary Corollary 1 will be fulfilled if we take any set of Lindblad operators contained exclusively in either symmetry sector. This will construct a dark Hamiltonian. For instance, in contrast to the main text, we may take a set of ultra-local spin dephasing Lindblad operators $L_k = \gamma_k S_k^z$ (and set a constant lattice potential ε_j=0). The difference to the example in the main text is that the stationary state is now given as,

$$\rho_\infty = C \exp\left(\beta_0 \eta^z + \beta_1(\eta^+\eta^-) + \beta_2 S^z\right), \tag{16}$$

where β_m play the role of chemical potentials of the grand canonical ensemble, and C is a normalization constant. This dissipation also constructs a dark Hamiltonian with the following eigenmodes,

$$\mathfrak{H}\left((\eta^+)^m \rho_\infty (\eta^-)^n\right) = 2(m-n)\mu\left((\eta^+)^m \rho_\infty (\eta^-)^n\right), \tag{17}$$

where μ is the chemical potential. Thus starting from an initial state with a well-defined number of particles and doublons we arrive at the stationary state given in Supplementary Equation (16). However, if we start from an initial state that is off-diagonal in both particle number and the number of doublons, we will get oscillations of the form of Supplementary Equation (17). The eigenmodes of the dark Hamiltonian all have η-pairing symmetry, which is of particular interest when studying superfluidity and superconductivity as η-paired states have long-range off-diagonal order[14] for all dimensions D. Thus all the eigenmodes of the dark Hamiltonian are superconductive.[14]

Transport of doublons

Let us now take Lindblad jump operators that drive doublons, i.e. in one spatial dimension $L_1 = \gamma_1 \eta_1^+$ and $L_2 = \gamma_2 \eta_n^-$, and an analogously chosen pair in higher spatial dimension.

For the same reasons as in the example in the main text we obtain a dark Hamiltonian. The crucial difference now is that the stationary state ρ_∞ will be different. As we are driving doublons, the eigenstates of the dark Hamiltonian will be *non-equilibrium* mixed states, which support a doublon current in addition to having coherent oscillations in the spin sector.

Experimental implementation with ultracold atoms

Ultracold fermionic atoms trapped in an optical lattice provide a clean experimental realization of the Hubbard model.[15] The dynamics of the atoms can be restricted to two atomic hyperfine states that realize

the two spin states of the Hubbard model. A magnetic field B is used to energetically split the hyperfine levels via the Zeeman effect. Tunneling between lattice sites τ is controlled by laser properties and short-ranged on-site fermion-fermion interactions U arise because of s-wave scattering between atoms in different spin states. Importantly, the thermal energy of ultracold atoms as well as the energy scales associated with τ, U and B can all be chosen to be much smaller than the gap between Bloch bands hence limiting the dynamics to a single band Hubbard model.

When immersing the optical lattice into a Bose-Einstein condensate additional scattering processes between the lattice atoms and the background Bose-Einstein condensate occur.[16] The dominant effect of these additional interactions is local pure dephasing γ_j of the lattice atoms described by the operators L_j in the main text (independently of whether the lattice atoms are bosons or fermions). The background gas may also cause small renormalizations of the parameters appearing in the Hubbard model but these are often negligible. Experimentally, such interactions have recently been studied for individual ^{133}Cs atoms immersed in ^{87}Rb condensates paving the way to controlled atom-quantum bath interactions.[17]

Our setup requires that the dephasing interaction affects both hyperfine states of the fermionic lattice atoms equally. This spin-agnostic interaction can for instance be realized by forming the Bose-Einstein condensate out of spin-0 bosons. Such two-component mixtures have recently been experimentally realized with fermionic ^{87}Sr and spin-0 ^{88}Sr.[18] Also mixtures of fermionic ^{40}K atoms and bosonic ^{87}Rb were described using spin-independent Bose-Fermi interactions in Supplementary Reference 19. Finally, to realize the $\eta-$paring example a uniform potential lattice potential is required as was recently realized experimentally in Supplementary Reference 20.

Supplementary References

[1] Albert, V. V., & Jiang, L. Symmetries and conserved quantities in Lindblad master equations. *Phys. Rev. A* **89,** 022118 (2014).

[2] Bellomo, B., Giorgi, G.L., Palma, G.M., Zambrini, R. Quantum synchronization as a local signature of super- and subradiance. *Phys. Rev. A* **95,** 043807 (2017).

[3] Lee, T.E. & Sadeghpour, H. R. Quantum Synchronization of Quantum van der Pol Oscillators with Trapped Ions. *Phys. Rev. Lett.* **111,** 234101 (2013).

[4] Xu, M, et al. Synchronization of two ensembles of atoms. *Phys. Rev. Lett.* **113,** 154101 (2014).

[5] Albert, V. V., Bradlyn, B., Fraas, M., & Jiang L. Geometry and Response of Lindbladians. *Phys. Rev. X* **6,** 041031 (2016).

[6] Baumgartner, B. & Narnhofer, H. Analysis of quantum semigroups with GKS-Lindblad generators: II. General. *J. Phys. A: Math. Theor.* **41,** 395303 (2008).

[7] Karasik, R.I, et al. Criteria for dynamically stable decoherence-free subspaces and incoherently generated coherences. *Phys. Rev. A* **77,** 052301 (2008).

[8] Brooke, P.G., Cresser, J.D., & Patra, M.K. Decoherence-free quantum information in the presence of dynamical evolution. *Phys. Rev. A* **77,**, 062313 (2008).

[9] Wu, S.L., Wang, L.C., & Yi, X.X. Time-dependent Decoherence-Free Subspace. *J. Phys. A: Math. Theor.* **45,** 405305 (2012).

[10] Daley A. Quantum Trajectories and open many-body quantum systems, *Adv. Phys.* **63,** 77 (2014).

[11] Kraus, B., Büchler, H. P., Diehl, S., Kantian, A., Micheli, A., & Zoller, P. Preparation of entangled states by quantum Markov processes. *Phys. Rev. A* **78,** 042307 (2008).

[12] Fernández-Hurtado, V., Mur-Petit, J., García-Ripoll, J. J., & Molina, R. A. Lattice scars: surviving in an open discrete billiard. *New J. Phys.* **16,** 035005 (2014)

[13] Turner, C.J., et al. Weak ergodicity breaking from quantum many-body scars. *Nat. Phys.* **14,** 745 (2018).

[14] Yang, C.N. η-pairing and off-diagonal long-range order in a Hubbard model. *Phys. Rev. Lett.* **63,** 2144 (1989).

[15] Esslinger, T. Fermi-Hubbard Physics with Atoms in an Optical Lattice. *Annu. Rev. Condens. Matter Phys.* **1,** 129 (2010).

[16] Klein A., Bruderer, M., Clark, S.R., & Jaksch D. Dynamics, dephasing and clustering of impurity atoms in Bose-Einstein condensates. *New J. Phys.* **9,** 411 (2007).

[17] D. Mayer, et al. Controlled doping of a bosonic quantum gas with single neutral atoms. Preprint at http://arxiv.org/abs/1805.01313 (2018).

[18] Mickelson, P.G., et al. Bose-Einstein condensation of ^{88}Sr through sympathetic cooling with ^{87}Sr. *Phys. Rev. A* **81,** 051601(R) (2010).

[19] Wang, D.-W., Lukin, M.D., Demler, E. Engineering Superfluidity in Bose-Fermi Mixtures of Ultracold Atoms. *Phys. Rev. A* **72,** 051604(R) (2005)

[20] Gaunt, A.L., et al. Bose-Einstein condensation of atoms in a uniform potential. *Phys. Rev. Lett.* **110,** 200406 (2013).
